# A comparative evaluation of commercially available non-herbal denture adhesives and essential oil based herbal denture adhesives on patient comfort and satisfaction- a clinical trial

**DOI:** 10.1186/s12903-025-06738-0

**Published:** 2025-08-23

**Authors:** Pooja Chitlange, Seema Sathe, Surekha Godbole, Aashika Agrawal

**Affiliations:** Department of Prosthodontics and Crown and Bridge, Sharad Pawar Dental College, Datta Meghe Institute of Higher Education and Research, Sawangi mehge, Wardha, Maharashtra India

**Keywords:** Complete denture, Denture adhesive, Herbal denture adhesive, Essential oil

## Abstract

**Background:**

Edentulism, the absence or total loss of all-natural teeth, is a common condition. Severe resorption of the residual alveolar ridge is one of the most common problems among edentulous patients, and it can still happen even with careful prosthetic therapy. Thus, the stability and retention of fully edentulous patients utilizing complete denture prostheses is one of the most significant challenges in dentistry today. Denture adhesive was used to solve this issue. According to studies, denture adhesive increases the surface tension between the denture fitting surface and the alveolar mucosa, improving stability and efficacy for those wearing complete dentures. Denture adhesive must be affordable, safe, and possess enough antimicrobial and antifungal qualities. It should have a pleasant fragrance, simple to apply, and not messy. It shouldn’t change or block the intaglio surface of the denture. Denture adhesives are currently recognized as therapies that can be applied in conjunction with dentures.

**Methods:**

On the Basis of Denture adhesive, the patients were randomly divided into two groups i.e. 20 patients in each group. Group A (commercially available denture adhesive i.e. Fixon) and Group B (Cinnamon oil incorporated denture adhesive). The patients were requested to fill out the questionnaire for masticatory efficiency, retention, stability, etc. at the time of denture insertion without using denture adhesive and seven days after the use of denture with denture adhesive.

**Results:**

The comparison between Non-Herbal and Herbal denture adhesive suggests comparable effectiveness in most aspects of denture satisfaction. However, Group B (Herbal Methods) showed significantly better outcomes in speech clarity (*p* = 0.047) and denture fit/removal (*p* = 0.037). These findings indicate a potential advantage of herbal methods in enhancing speech and ease of denture use.

**Conclusions:**

Elderly patients benefit from denture adhesives for improved comfort and confidence. This study found comparable satisfaction between herbal and non-herbal adhesives, with herbal methods showing significantly better results in speech clarity and denture fit/removal. Herbal adhesives may offer added advantages, warranting further research with larger sample sizes.

**Trial registration:**

: The study is registered with the Clinical Trial Registry-India under the CTRI Registration number CTRI/2024/06/068933, registered on June 14, 2024.

**Supplementary Information:**

The online version contains supplementary material available at 10.1186/s12903-025-06738-0.

## Background

The lack or complete loss of natural teeth is known as edentulism, which happens frequently. Edentulous patients frequently experience severe alveolar ridge atrophy, which can happen even with careful prosthesis treatment. The stability and retention of the prosthesis in the rehabilitation of those who are entirely edentulous are therefore among the main challenges in dentistry. To improve denture adherence and retention, dentists use denture adhesive [1]. According to studies, denture adhesive increases the surface tension between the denture fitting surface and the alveolar mucosa, improving stability and efficacy for those wearing complete dentures. Denture adhesive must be affordable and possess antimicrobial and antifungal qualities. It should be easy to maintain hygienic standards, be user-friendly, and possess an appealing taste and aroma. The denture’s intaglio surface shouldn’t be altered or harmed [2]. Additionally, it gives the patient a sense of security and confidence [3].

Denture adhesives are categorized according to various forms, including paste, gels, tapes, and powders. Even in cases when the denture is ill-fitting, denture adhesive improves retention, reduces pain, prevents strangling the mucosal blood supply, and reduces the need for adjustments [4]. Improvements in biting force and chewing efficiency have been reported; this results in a decrease in local pressure and a greater distribution of occlusal forces over the tissues supporting the denture. Adhesives’ lubricating and cushioning qualities reduce mucosal friction and pain. Patients with muscular control impairments brought on by hormonal or neurotransmitter problems may find great benefit from denture adhesives [5].

In this study Fixon denture adhesive was selected group as it is easily available, economic and have less adverse effects on oral tissues. Nanoparticles of Cinnamon oil (essential oil) can be easily incorporated in the Fixon denture adhesive.

## Methods

### Aim

This study aims to evaluate and compare indigenous herbal denture adhesive with and without essential oil in terms of patient satisfaction with denture retention, stability, and masticatory efficiency.

#### Objectives

- To assess the patient’s satisfaction with denture retention at the time of denture insertion without using Denture adhesive.
- To assess the patient’s satisfaction with the herbal denture adhesive on retention of dentures after 7 days of use by the patient.
- To assess the patient’s satisfaction with herbal denture adhesive incorporated with essential oil on retention of the denture after 7 days of its use by the patient.
- Comparative evaluation of the patient’s satisfaction with retention of dentures after using herbal denture adhesive with and without incorporation of essential oil.

### Settings and locations where the data were collected

This randomized controlled trial was conducted in the Department of Prosthodontics, SPDC&H, Sawangi (M), Wardha. The data was collected in the postgraduate clinic of the Department of Prosthodontics.

### Eligibility criteria for participants

#### Inclusion criteria

- Patients who are old denture wearers.
- Patients with complete denture prosthesis.
- Patients with severely resorbed ridges.

#### Exclusion criteria

- Patients with tooth support overdenture.
- Patients with implants supported overdentures.
- Patients with a single complete denture.

Based on the inclusion criteria 40 patients were selected from the out-patient department of Prosthodontics and they will be divided randomly into two groups: Group A (*n* = 20) (commercially available denture adhesive i.e. Fixon) and Group B (*n* = 20) (denture adhesive with Cinnamon oil incorporated herbal denture adhesive). Patients were requested to fill out the questionnaire for masticatory efficiency, retention, stability, etc. of the denture at the time of denture insertion without using denture adhesive and 7 days after using denture adhesive.

To gather information about denture fitting, removal, chewing, retention, and phonetics, a standard questionnaire form was given to each patient. Following the patient’s answer to Questionnaire 1 [1, 6, 7], a clinical examination and application of denture adhesive were demonstrated to patients, with the manufacturer’s recommendations being followed. Patients were explained about the application and cleaning of denture adhesive for the next 7 days. The patients were recalled a week later to complete an additional questionnaire 2 [1, 6, 7] in which they were asked to rate the denture’s strength, biocompatibility, convenience, and masticatory abilities using the denture adhesive that was provided.

### Preparation of indigenous herbal adhesive

Formulating a denture adhesive that incorporates cinnamon oil as an essential oil in powder form can be a unique challenge, as essential oils are typically liquids. However, it can be incorporated by using the active compounds of cinnamon oil into a powdered form, using a suitable carrier to encapsulate or stabilize the oil.

### Procedure

To synthesize mesoporous silica nanoparticles (MSN), 0.78 g of CTAB (Cetyltrimethylammonium Bromide) is dissolved in 21.6 mL of nano pure water, followed by the addition of 3.42 mL of ethanol and 41.02 µL of diethanolamine (DEA). The mixture is stirred for 30 min at 60 °C. Then, 2.1 g of TMOS (Tetramethyl orthosilicate) is added slowly and stirred for 2 h. After washing with methanol and ethanol, 1.44 mL of HCl is added for CTAB removal, stirring for 6–8 h. 2 mL of 5% cinnamon oil is introduced. Finally, after washing and drying the MSN, 1 g of commercially available denture adhesive (Fixon) is incorporated, and the product is washed and dried.

### Application of both denture adhesive

To apply denture adhesive, first it was ensured that the dentures are clean and dry. The powder was sprinkled onto the dampened denture base, covering it evenly with a light dusting, focusing on the areas that contacted the gums. A few drops of water were added, and the denture was inserted into the mouth and pressed gently for a few seconds to allow the adhesive to bond. A waiting period of 5–10 min was observed to let the adhesive set before eating or drinking. After use, the dentures were removed gently, and both the dentures and gums were cleaned to remove any remaining adhesive. Reapplication was done as needed throughout the day.

Patients were advised to wear their dentures throughout the day and remove them at night or whenever not in use, storing them in water to prevent drying. They were also instructed to clean both the dentures and oral mucosa thoroughly to ensure complete removal of adhesive. Dentures should be gently brushed after each use with a soft-bristled brush and a suitable denture cleanser.

### Sample size

The sample size is determined using the following formula,

$$n\;=\;(Z\;1-\alpha/2\;+\;Z\;1-\;\beta)2\;p1(1-p1)\:+\:p2\;(1-\;p2).\;\;\;\;\;\;\;\;\;(p1-\;p2)2$$

Proportion of outcome from group 1 (p_1_) = 0.37.

Proportion of outcome from group 2 (p_2_) = 0.22.

Level of significance (α) = 0.05.

Power (_1−_ β) = 0.80.

Z alpha value = 1.96.

Z beta value = 0.84.

Total Sample size = 40.

Sample size estimation is done at a 95% confidence interval. Each group consist of 20 samples.

### Statistical analysis

This study examines the effectiveness of non-herbal and herbal groups in improving denture satisfaction among participants. A paired t-test was conducted to compare pre-test and post-test responses across multiple aspects of denture use, including retention, comfort, chewing ability, speech, fit, and soft tissue impact. An independent t-test is used for post-test comparisons between two groups. A total of 20 participants were assessed before and after intervention using a satisfaction questionnaire. Mean scores, standard deviations, t-values, and p-values were calculated to determine statistically significant improvements (*p* < 0.05) in denture satisfaction.

## Results

The comparison of pre- and post-test scores (Table 1) revealed notable improvements in several aspects of denture satisfaction with non-herbal adhesive use. Significant enhancements were observed in upper denture retention (*p* = 0.014), chewing ability (*p* = 0.012), fit and removal (*p* = 0.002), and swallowing comfort (*p* < 0.001). Lower denture retention and comfort improved but were not statistically significant. Soft tissue health also improved, though the change was marginally non-significant (*p* = 0.062), and no soreness or inflammation was reported. However, speech and sound production showed a statistically significant decline (*p* = 0.004), possibly due to adaptation issues with new dentures. These findings indicate that non-herbal adhesives effectively enhance key functional aspects of denture use, especially retention, chewing, and swallowing. While improvements in comfort and soft tissue health were noted, they were not statistically significant, highlighting the need for further long-term studies. Speech adaptation remains a concern and warrants targeted strategies in future interventions.

**Table 1:** Shows the pre-and post-comparison of non-herbal methods

QUESTIONS	group	N	Mean	Std. Deviation	t value	P value	MEAN Difference
To what extent do you find the retention of your upper denture satisfactory?	pre-test	20	1.9500	0.75915	6.613	0.014	− 0.90000
	post-test	20	2.8500	0.36635			
To what extent do you find the retention of your lower denture satisfactory?	pre-test	20	1.5500	0.68633	0.041	0.841	−1.00000
	post-test	20	2.5500	0.68633			
To what extent do you find the chewing ability of your denture satisfactory?	pre-test	20	1.2500	0.44426	6.915	0.012	−1.65000
	post-test	20	2.9000	0.30779			
To what extent you are satisfied with the comfort of your new denture?	pre-test	20	1.5000	0.68825	3.137	0.085	− 0.55000
	post-test	20	2.0500	0.60481			
To what extent do you find the speech and sound produced by the denture satisfactory?	pre-test	20	2.1500	0.74516			0.30000
	post-test	20	1.8500	0.36635	9.309	0.004	
How satisfied are you with the way your dentures fit and removal?	pre-test	20	1.5500	0.68633	11.097	0.002	− 0.65000
	post-test	20	2.2000	0.41039			
Does your denture harm the soft tissues in any way?	pre-test	20	1.7500	0.44426	3.709	0.062	− 0.65000
	post-test	20	2.4000	0.50262			
Do you have any issues with swallowing because of the denture?	pre-test	20	1.8500	0.36635	16.850	0.000	− 0.60000
	post-test	20	2.4500	0.51042			

The comparison of pre- and post-test scores (Table 2) indicates that herbal denture adhesives significantly improved upper denture retention (*p* = 0.003), chewing ability (*p* < 0.001), comfort (*p* = 0.034), and soft tissue health (*p* = 0.001). Chewing ability showed the most substantial improvement, followed by soft tissue harm reduction. While lower denture retention, speech clarity, fit and removal, and swallowing comfort also showed positive trends, these changes were not statistically significant. The mean scores for these parameters increased, suggesting potential benefits, but further validation with a larger sample is needed. These findings suggest that herbal methods can effectively enhance key aspects of denture satisfaction, especially functional performance and oral tissue comfort. However, areas such as speech, swallowing, and ease of denture handling may require targeted strategies or longer adaptation periods. Continued research is recommended to establish the full potential of herbal adhesives in improving overall denture experience in elderly patients (Table 3).

**Table 2:** Shows the pre and post-comparison of herbal methods

QUESTIONS	group	N	Mean	Std. Deviation	P value	t value	MEAN Difference
To what extent do you find the retention of your upper denture satisfactory?	pre-test	20	1.9000	0.78807	0.003	−4.889	− 0.95000
	post-test	20	2.8500	0.36635			
To what extent do you find the retention of your lower denture satisfactory?	pre-test	20	1.7500	0.78640	2.966	0.093	− 0.90000
	post-test	20	2.6500	0.58714			
To what extent do you find the chewing ability of your denture satisfactory?	pre-test	20	1.4500	0.68633	19.134	0.000	−1.45000
	post-test	20	2.9000	0.30779			
To what extent you are satisfied with the comfort of your new denture?	pre-test	20	1.6000	0.75394	4.844	0.034	− 0.45000
	post-test	20	2.0500	0.60481			
To what extent do you find the speech and sound produced by the denture satisfactory?	pre-test	20	2.0000	0.79472	2.068	0.159	− 0.75000
	post-test	20	2.7500	0.55012			
How satisfied are you with the way your dentures fit and removal?	pre-test	20	1.6000	0.68056	3.054	0.089	− 0.80000
	post-test	20	2.4000	0.50262			
Does your denture harm the soft tissues in any way?	pre-test	20	1.8500	0.36635	13.345	0.001	− 0.55000
	post-test	20	2.4000	0.50262			
Do you have any issues with swallowing because of the denture?	pre-test	20	1.8000	0.41039	2.083	0.157	− 0.50000
	post-test	20	2.3000	0.47016			

**Table 3:** Post-Test results for group A (Non-Herbal Methods) and group B (Herbal Methods)

QUESTIONS	*N*	Mean	Std. Deviation	Mean	Std. Deviation	*P* value
		GROUP A		GROUP B		
Is retention of the upper denture satisfactory?	20	2.8500	0.36635	2.8500	0.36635	0.471
Is retention of lower dentures satisfactory?	20	2.5500	0.68633	2.6500	0.58714	0.384
Chewing ability satisfactory?	20	2.9000	0.30779	2.9000	0.30779	0.338
Comfort of the new denture satisfactory?	20	2.0500	0.60481	2.0500	0.60481	0.483
Speech and sound satisfactory?	20	1.8500	0.36635	2.7500	0.55012	0.047*
Satisfaction with denture fit and removal?	20	2.2000	0.41039	2.4000	0.50262	0.037*
Denture harming soft tissues?	20	2.4000	0.50262	2.4000	0.50262	0.583
Issues with swallowing due to denture?	20	2.4500	0.51042	2.3000	0.47016	0.620

No significant difference was observed between Group A and Group B for upper denture retention (*p* = 0.471) and lower denture retention (*p* = 0.384). Both groups reported similar levels of satisfaction. Chewing ability and comfort were identical in both groups (Mean = 2.9000 for chewing; Mean = 2.0500 for comfort), with no statistically significant difference (*p* = 0.338 and *p* = 0.483, respectively). Speech and sound satisfaction showed a statistically significant difference (*p* = 0.047), indicating that Group B (Herbal Methods) had higher satisfaction in speech clarity compared to Group A. Satisfaction with denture fit and removal was significantly higher in Group B (*p* = 0.037), suggesting that herbal methods might provide better adaptation and ease of use. Soft tissue impact and swallowing issues showed no significant difference between the two groups (*p* = 0.583 and *p* = 0.620, respectively). This indicates that both methods had similar effects on tissue health and swallowing comfort.

The post-test comparison between Non-Herbal and Herbal groups suggests comparable effectiveness in most aspects of denture satisfaction. However, Group B (Herbal Methods) showed significantly better outcomes in speech clarity (*p* = 0.047) and denture fit/removal (*p* = 0.037). These findings indicate the herbal denture adhesive with cinnamon oil performed well due to its antimicrobial and anti-inflammatory properties, which enhance oral comfort and reduce irritation. Its natural bio-adhesive effect improves retention, while the pleasant flavor promotes patient compliance. These factors likely contributed to better chewing ability, comfort, and soft tissue health.

## Discussion

The present study aimed to evaluate and compare the effectiveness of herbal and non-herbal denture adhesive on various aspects of denture satisfaction among edentulous patients. Both groups showed improvements across multiple domains; however, specific differences emerged that highlight the unique strengths of each approach.

Significant improvements were observed in upper denture retention, chewing ability, and swallowing comfort in both groups. These findings were similar with previous studies such as that of Mekkawy et al. [1], where commercial denture adhesives (COREGA and SECURE) improved mandibular retention and chewing ability. Similarly, our study found enhanced retention, especially in the upper denture, following both herbal and non-herbal denture adhesive applications, with statistical significance noted in the non-herbal group (*p* = 0.014) and moderate significance in the herbal group (*p* = 0.003).

Chewing ability exhibited a highly significant increase in both groups, which is consistent with results reported by Koronis et al. [8], who noted that adhesives improved chewing function and patient satisfaction, particularly among patients with poor baseline retention. The chewing scores in our study improved markedly in the herbal group (*p* < 0.001) and significantly in the non-herbal group (*p* = 0.012), confirming the value of both interventions in enhancing oral function.

Soft tissue health improved significantly in the herbal group (*p* = 0.001), suggesting a unique benefit of herbal formulations, possibly due to their anti-inflammatory or soothing properties. This finding was similar with the results of Yadav et al. [7], who demonstrated that herbal components like Isabgol improved retention and oral comfort more effectively than some commercial adhesives. Conversely, in the non-herbal group, while soft tissue harm reduction was observed, the result did not reach statistical significance (*p* = 0.062), indicating that herbal interventions may be more beneficial in this area.

Comfort of the new dentures improved in both groups, although it was statistically significant only in the herbal group (*p* = 0.034). In contrast to Ereifej et al. [9]., who reported substantial improvements in patient satisfaction with commercial adhesives across all categories, our study suggests that herbal methods may offer comparable if not superior comfort, potentially due to their biocompatibility and fewer chemical irritants.

Satisfaction with fit and removal was significantly better in the herbal group (*p* = 0.037), supporting the notion that herbal adhesives or treatments might offer better manageability, echoing the findings of Koronis et al., who found Fittydent to be most effective for patient satisfaction and usability [8].

The primary drawback of denture adhesives is the risk of over-reliance, which can conceal issues related to poor denture fit, such as improper occlusion, ridge resorption, or prosthesis wear. This may result in patients continuing to use ill-fitting dentures, potentially leading to soft tissue irritation, infections, and progressive bone loss. Long-term use may also be associated with difficulties in cleaning and an unpleasant taste or texture, affecting overall oral hygiene and comfort.

Overall, both herbal and non-herbal interventions were effective in improving denture satisfaction. However, the herbal method showed particular promise in enhancing speech, comfort, and soft tissue health. These findings underscore the potential of herbal treatments as viable alternatives or complements to conventional adhesives. Further research with larger sample sizes and longer follow-up periods is recommended to validate and expand upon these outcomes.

## Conclusions

Elderly patients often face physical and psychological challenges when adapting to new or relined dentures. Denture adhesives can play a crucial role in improving comfort, fit, and overall satisfaction. Based on the present findings, both herbal and non-herbal denture adhesives demonstrated comparable effectiveness across most parameters of denture satisfaction. However, Group B (Herbal Methods) showed significantly better outcomes in terms of speech clarity (*p* = 0.047) and ease of denture fit and removal (*p* = 0.037). These results suggest that herbal adhesives may offer specific advantages in improving functional aspects such as speech and handling, potentially enhancing the patient’s confidence and adaptation to the prosthesis.

### Limitations

In the given sample, the herbal denture adhesive showed statistically significant results. However, potential disadvantages of denture adhesives should be carefully considered. Further studies with larger sample sizes are recommended to validate these findings and ensure long-term safety and effectiveness.

## Supplementary Information

Supplementary Material 1.

## Data Availability

All the required data is provided within the manuscript. for further data contact to corresponging author. All the data is available with Corresponding Author.
