# Supplementary material for: A comparative evaluation of commercially available non-herbal denture adhesives and essential oil based herbal denture adhesives on patient comfort and satisfaction- a clinical trial
Source: BMC Oral Health. 2025 Aug 23;25:1357. doi: 10.1186/s12903-025-06738-0 (PMC12374332; doi:10.1186/s12903-025-06738-0)
Supplement: Supplementary file 1 — Supplementary Material 1. [file 12903_2025_6738_MOESM1_ESM.docx]

Table 1: shows the pre-and post-comparison of non-herbal methods

| Table 1: Pre-post comparison of non-herbal methods | | | | | |  |  |
| --- | --- | --- | --- | --- | --- | --- | --- |
| QUESTIONS | group | N | Mean | Std. Deviation | t value | P value | MEAN Difference |
| To what extent do you find the retention of your upper denture satisfactory? | pre-test | 20 | 1.9500 | .75915 | 6.613 | .014 | -.90000 |
|  | post-test | 20 | 2.8500 | .36635 |  |  |  |
| To what extent do you find the retention of your lower denture satisfactory? | pre-test | 20 | 1.5500 | .68633 | .041 | .841 | -1.00000 |
|  | post-test | 20 | 2.5500 | .68633 |  |  |  |
| To what extent do you find the chewing ability of your denture satisfactory? | pre-test | 20 | 1.2500 | .44426 | 6.915 | .012 | -1.65000 |
|  | post-test | 20 | 2.9000 | .30779 |  |  |  |
| To what extent you are satisfied with the comfort of your new denture? | pre-test | 20 | 1.5000 | .68825 | 3.137 | .085 | -.55000 |
|  | post-test | 20 | 2.0500 | .60481 |  |  |  |
| To what extent do you find the speech and sound produced by the denture satisfactory? | pre-test | 20 | 2.1500 | .74516 |  |  | .30000 |
|  | post-test | 20 | 1.8500 | .36635 | 9.309 | .004 |  |
| How satisfied are you with the way your dentures fit and removal? | pre-test | 20 | 1.5500 | .68633 | 11.097 | .002 | -.65000 |
|  | post-test | 20 | 2.2000 | .41039 |  |  |  |
| Does your denture harm the soft tissues in any way? | pre-test | 20 | 1.7500 | .44426 | 3.709 | .062 | -.65000 |
|  | post-test | 20 | 2.4000 | .50262 |  |  |  |
| Do you have any issues with swallowing because of the denture? | pre-test | 20 | 1.8500 | .36635 | 16.850 | .000 | -.60000 |
|  | post-test | 20 | 2.4500 | .51042 |  |  |  |

Table 2: Shows the Pre and post-comparison of herbal methods

| Table 2: Pre-post comparison of herbal methods | | | | | |  |  |
| --- | --- | --- | --- | --- | --- | --- | --- |
| QUESTIONS | group | N | Mean | Std. Deviation | P value | t value | MEAN Difference |
| To what extent do you find the retention of your upper denture satisfactory? | pre-test | 20 | 1.9000 | .78807 | .003 | -4.889 | -.95000 |
|  | post-test | 20 | 2.8500 | .36635 |  |  |  |
| To what extent do you find the retention of your lower denture satisfactory? | pre-test | 20 | 1.7500 | .78640 | 2.966 | .093 | -.90000 |
|  | post-test | 20 | 2.6500 | .58714 |  |  |  |
| To what extent do you find the chewing ability of your denture satisfactory? | pre-test | 20 | 1.4500 | .68633 | 19.134 | .000 | -1.45000 |
|  | post-test | 20 | 2.9000 | .30779 |  |  |  |
| To what extent you are satisfied with the comfort of your new denture? | pre-test | 20 | 1.6000 | .75394 | 4.844 | .034 | -.45000 |
|  | post-test | 20 | 2.0500 | .60481 |  |  |  |
| To what extent do you find the speech and sound produced by the denture satisfactory? | pre-test | 20 | 2.0000 | .79472 | 2.068 | .159 | -.75000 |
|  | post-test | 20 | 2.7500 | .55012 |  |  |  |
| How satisfied are you with the way your dentures fit and removal? | pre-test | 20 | 1.6000 | .68056 | 3.054 | .089 | -.80000 |
|  | post-test | 20 | 2.4000 | .50262 |  |  |  |
| Does your denture harm the soft tissues in any way? | pre-test | 20 | 1.8500 | .36635 | 13.345 | .001 | -.55000 |
|  | post-test | 20 | 2.4000 | .50262 |  |  |  |
| Do you have any issues with swallowing because of the denture? | pre-test | 20 | 1.8000 | .41039 | 2.083 | .157 | -.50000 |
|  | post-test | 20 | 2.3000 | .47016 |  |  |  |

Table 3: Post-Test Results for Group A (Non-Herbal Methods) and Group B (Herbal Methods)

| QUESTIONS | N | Mean | Std. Deviation | Mean | Std. Deviation | P value |
| --- | --- | --- | --- | --- | --- | --- |
|  |  | GROUP A | | GROUP B | |  |
| Is retention of the upper denture satisfactory? | 20 | 2.8500 | 0.36635 | 2.8500 | 0.36635 | 0.471 |
| Is retention of lower dentures satisfactory? | 20 | 2.5500 | 0.68633 | 2.6500 | 0.58714 | 0.384 |
| Chewing ability satisfactory? | 20 | 2.9000 | 0.30779 | 2.9000 | 0.30779 | 0.338 |
| Comfort of the new denture satisfactory? | 20 | 2.0500 | 0.60481 | 2.0500 | 0.60481 | 0.483 |
| Speech and sound satisfactory? | 20 | 1.8500 | 0.36635 | 2.7500 | 0.55012 | 0.047* |
| Satisfaction with denture fit and removal? | 20 | 2.2000 | 0.41039 | 2.4000 | 0.50262 | 0.037* |
| Denture harming soft tissues? | 20 | 2.4000 | 0.50262 | 2.4000 | 0.50262 | 0.583 |
| Issues with swallowing due to denture? | 20 | 2.4500 | 0.51042 | 2.3000 | 0.47016 | 0.620 |
